# Análise de sobrevivência em estudos clínicos e experimentais

**DOI:** 10.1590/1677-5449.001604

**Published:** 2017

**Authors:** Hélio Amante Miot

**Affiliations:** 1 Universidade Estadual Paulista – UNESP, Faculdade de Medicina de Botucatu, Departamento de Dermatologia e Radioterapia, Botucatu, SP, Brasil.

Usualmente, os desfechos de um estudo são representados pela frequência de um evento categórico (por exemplo, mortalidade, cura, fechamento da ferida) ou pela intensidade de um fenômeno mensurado quantitativamente (por exemplo, nível pressórico, fração de obstrução arterial, índice de qualidade de vida).

Entretanto, em certos estudos de seguimento longitudinal, interessa ao pesquisador avaliar o tempo demandado até a ocorrência de um evento (por exemplo, tempo até a reoclusão arterial, sobrevida livre de doença, tempo de incubação). Esse tipo de investigação apresenta uma particularidade: os sujeitos do estudo podem permanecer em observação por tempos distintos. Alguns deixam o estudo pela ocorrência do evento, porém outros perdem o seguimento por razões diferentes do desfecho (adoecem ou morrem de outras causas, retiram o consentimento, mudam de endereço, apresentam efeitos adversos graves, necessitam interromper o tratamento). Ou, ainda, o estudo se encerra. Para contemplar tais situações especiais, foram desenvolvidos modelos estatísticos agrupados pelo termo análise de sobrevivência, em que a variável dependente passa a ser o tempo até o evento, e os sujeitos são computados como pessoas*tempo^1^^,^ 2.

**Figura 1:**
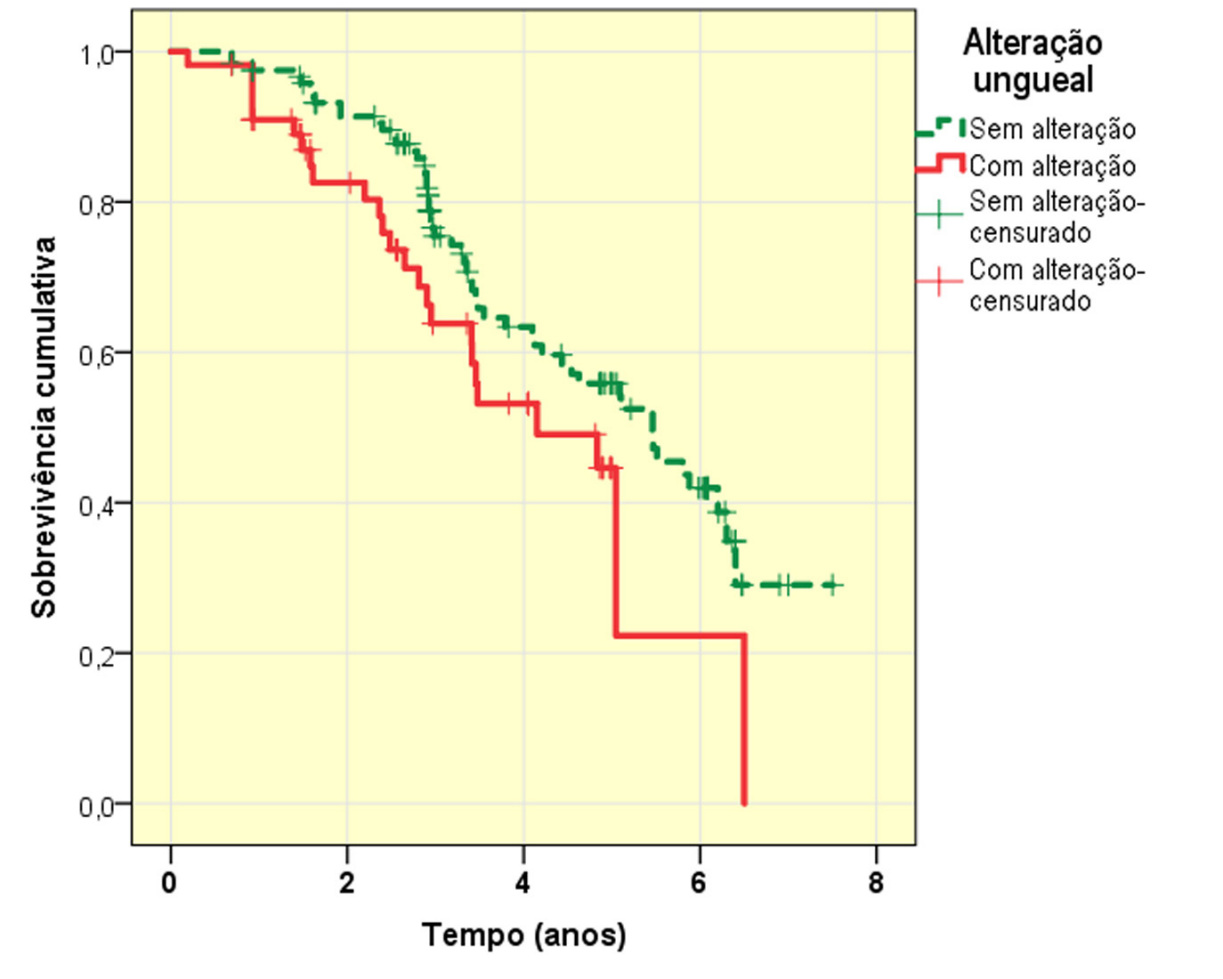
Funções de sobrevivência (curva de Kaplan-Meier) para eventos cardiovasculares em homens maiores de 55 anos (n = 176) provenientes do Hospital das Clínicas da Faculdade de Medicina de Botucatu (Botucatu, SP), de acordo com a alteração do ângulo ungueal de Lovibond (≥ 180º). Log-rank (p = 0,06); Tarone-Ware (p = 0,05); Gehan-Breslow (p = 0,04); Peto-Prentice (p = 0,04).

Os dados da análise de sobrevivência podem ser representados pela curva de sobrevivência (Kaplan-Meier) e pela tabela de sobrevivência, que, em função do tempo, refletem a fração de sujeitos que permanecem em observação, ou seja, não sofreram o evento nem foram “censurados”, termo usado para a interrupção do seguimento (Figura 1). A partir dessas análises, pode-se estimar parâmetros como o tempo até atingir um percentual de desfechos e o percentual de eventos que ocorrem em um intervalo de tempo, ou fazer uma comparação dos tempos para ocorrência dos eventos nos diferentes subgrupos^3^^-^ 5.

A título de ilustração, considere-se uma coorte de 176 homens maiores de 55 anos, seguidos por 10 anos (estudo ainda em andamento), para se avaliar a ocorrência de eventos cardiovasculares (infarto do miocárdio, angina, claudicação, acidente vascular cerebral, cirurgia de revascularização arterial) e sua associação com alterações cutâneas, neste exemplo a planificação do ângulo ungueal de Lovibond (≥ 180º). Em um tempo mediano (p25-75) de seguimento de 3,2 (2,5-5,0) anos, houve 25 eventos (45%) entre os portadores de alteração ungueal e 53 eventos (44%) entre os não portadores (RR = 1,01, IC95% 0,65 a 1,57; p = 0,95). Entretanto, em função do tempo, os eventos ocorreram mais precocemente entre os portadores de alteração ungueal (Figura 1). Aos quatro anos de seguimento, metade dos casos já haviam interrompido a observação, enquanto mais de 60% dos controles ainda participavam do estudo, e que somente atingiram 50% de sobrevida após 5 anos de seguimento. A probabilidade de sobrevivência após um tempo de seguimento ou a regularidade das taxas de mortalidade também podem ser estimadas.

Os principais testes de hipóteses para a comparação inferencial dos subgrupos são: Gehan-Breslow (Wilcoxon generalizado) e Peto-Prentice, que estabelecem pesos maiores para o maior número de casos sob risco (eventos no início da observação); Tarone-Wire, que pondera tanto os pesos do número de casos como o tempo de observação (sensível aos eventos do meio da observação); e Log-rank (Mantel-Cox), no qual todos os pontos de observação apresentam o mesmo peso, favorecendo as diferenças observadas ao final do seguimento^6^^,^ 7. Todos os testes perdem poder se as taxas de eventos se alternarem entre os grupos em função do tempo (cruzamento das linhas).

A partir do exemplo da Figura 1, como há muitas censuras e eventos já na primeira metade do seguimento, os testes Gehan-Breslow, Peto-Prentice e Tarone-Ware indicaram valores de p ≤ 0,05. Já o teste Log-rank foi influenciado pela segunda metade do seguimento, com menor número de casos em observação, resultando em significância marginal (p = 0,06). Mais do que a busca por significância estatística, o pesquisador deve ser criterioso na escolha e interpretação dos testes, visando a generalização dos resultados, até porque a ausência de eventos em um subgrupo, associada a uma grande frequência de censuras, deve alertar para possíveis razões de descontinuidade associadas à exposição^5^^,^ 8.

A dimensão do efeito entre os subgrupos em uma análise de sobrevivência é estimada pelo *hazard ratio* (HR), que pode ser interpretado como o risco relativo da ocorrência do evento em função do tempo. O cálculo do HR é fornecido a partir do modelo de riscos proporcionais (regressão de Cox), que permite ainda o ajuste do HR para outras covariáveis (independentemente da distribuição), fornecendo uma análise multivariada do estudo^2^^,^ 9 ^,^ 10.

A representação do HR deve acompanhar sua estimativa de 95% e o valor de p. No exemplo da Figura 1, após ajuste para idade, tabagismo, dislipidemia, diabetes, história familiar e hipertensão, o HR da alteração ungueal foi 1,7 (IC 95% 1,1 a 2,9; p = 0,03). A interpretação é de que, para a população estudada, eventos cardiovasculares ocorreram 1,7 vez mais rapidamente nos portadores de alterações ungueais, com diferença significativa, independentemente dos demais fatores de risco clássicos^11^^-^ 13.

**Figura 2:**
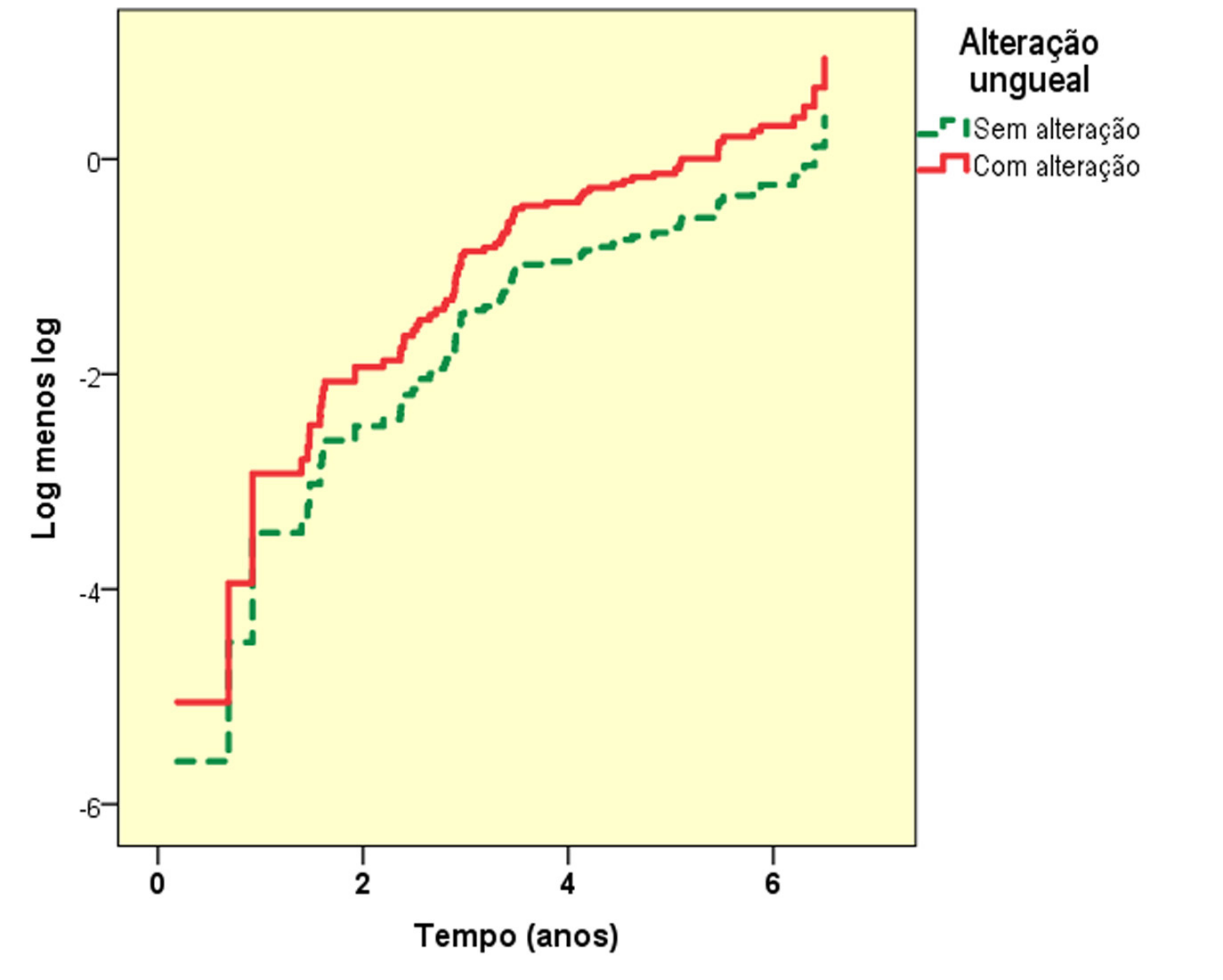
Diagrama Log-Log dos dados da análise de sobrevivência para eventos cardiovasculares em homens maiores de 55 anos (n = 176) provenientes do Hospital das Clínicas da Faculdade de Medicina de Botucatu (Botucatu, SP), de acordo com a alteração do ângulo ungueal de Lovibond (≥ 180º).

A condição para o adequado desempenho do modelo de Cox é o paralelismo (homogeneidade do risco em função do tempo) entre a ocorrência de eventos dos subgrupos comparados; caso contrário, o HR variaria em função do tempo de seguimento. O principal método para avaliar tal paralelismo é o diagrama Log-Log (Figura 2), que não deve apresentar cruzamento entre as linhas^2^^,^ 8 ^,^ 11 ^,^ 13.

Muitas vezes, a variável dependente do estudo longitudinal é registrada como quantitativa (por exemplo, pressão arterial, nível glicêmico, índice de qualidade de vida). Nesses casos, é necessária a dicotomização ou a ordenação das variáveis (por exemplo, hipertenso, diabético, nível de impacto na qualidade de vida, obstrução arterial < 50%) para proceder à análise de sobrevivência. Os critérios de escolha dos pontos de corte para a categorização têm impacto direto nos resultados, devendo ser definidos com parcimônia e plausibilidade científica, além de justificados detalhadamente na metodologia. É também recomendável proceder à análise de sensibilidade dos resultados, ponderando o impacto de diferentes pontos de corte na dimensão dos resultados, a fim de aumentar a consistência das conclusões^14^.

O dimensionamento amostral para estudos longitudinais que utilizem análise de sobrevivência é influenciado pelo tempo de seguimento, número de censuras, número de subgrupos para comparação e frequência total e diferencial de eventos identificados entre os subgrupos. De forma geral, os modelos não costumam apresentar bom desempenho (maior erro tipo 2) quando ocorrem menos de 10 eventos (por subgrupo de análise) e o número de sujeitos for inferior a 10 por subgrupo. Partindo desses princípios, é recomendável a realização de um pré-teste em um tempo de seguimento abreviado a fim de se adequar a amostra^15^^,^ 16. Abaixo apresentamos uma fórmula que permite dimensionar o número de eventos necessários em função do HR e que depende da tolerância aos erros tipo 1, usualmente 5% bicaudal (Z_α/2_ = 1,96), e tipo 2, usualmente 20% unicaudal (Z_β_ = 0,84)^16^^,^ 17. A proporção de sujeitos em cada subgrupo é representado por p1 e p2.

$$Eventos={\left(Z_{\alpha /2}+Z_{\beta }\right)}^{2}/p1\times p2\times {\left(\ln\;HR\right)}^{2}$$ (1)

Considerando um pré-teste com os dados do exemplo da Figura 1, temos dois grupos: com 120 (68%) e 56 (32%) sujeitos. Houve 78 eventos e identificou-se um HR de 1,7. Aplicando esses dados à fórmula acima, teremos: (1,96 + 0,84)^2^ / 0,32 × 0,68 × (*ln* 1,7)^2^ = 128 eventos necessários. Isso indica a necessidade de ampliação da amostra e/ou do tempo de seguimento.

Por ser uma técnica muito sensível à mudança, a análise de sobrevivência deve ser conduzida com máximo rigor metodológico, sendo recomendável o apoio de estatístico ou epidemiologista experiente. Vieses de seleção dos subgrupos, diferentes tempos de doença antes da alocação (censura à esquerda), irregularidades na randomização e falhas no registro ou no controle das censuras são exemplos metodológicos que comprometem os resultados. Casos especiais como a comparação de dados emparelhados, a ordenação dos fatores de comparação (por exemplo, estadiamento oncológico), covariáveis com comportamentos que se modificam em função do tempo (por exemplo, dose de medicamento, níveis de colesterol), comparação de grupos com comportamentos não paralelos, ou eventos recorrentes (por exemplo, reinfecção, reoclusão, reinfarto) demandam diferentes modelagens que extrapolam o escopo deste texto^5^^,^ 8.

Por fim, a escolha de uma técnica de análise de sobrevivência para a avaliação dos dados longitudinais não exclui outras modalidades clássicas de análise estatística no mesmo estudo, mas redimensiona a percepção do mesmo fenômeno^18^.
