# Supplementary material for: Análise de sobrevivência em estudos clínicos e experimentais
Source: J Vasc Bras. 2017 Oct-Dec;16(4):267–9. [Article in Portuguese] doi: 10.1590/1677-5449.001604 (PMC5944302; doi:10.1590/1677-5449.001604)
Supplement: Translation

# Translation: Survival analysis in clinical and experimental studies

**Authors:** Hélio Amante Miot

**Affiliations:** 1 Universidade Estadual Paulista – UNESP, School of Medicine, Department of Dermatology and Radiotherapy, Botucatu, SP, Brazil.

Study outcomes are usually expressed as the frequency of a categorical event (for example, mortality, cure, wound closure) or the intensity of a phenomenon measured quantitatively (for example, blood pressure, proportion of an artery obstructed, or a quality of life index).

However, in some longitudinal follow-up studies, researchers are interested in evaluating the time elapsed before an event occurs (for example, time until an artery is re-occluded, disease-free survival, incubation time). This type of investigation has a specific characteristic: different participants can be under observation for different periods of time. Some drop out of the study because the specific event has occurred, but others can be lost to follow-up for reasons other than the outcome of interest (they fall ill or die from other causes, withdraw their consent, change address, or exhibit serious adverse effects, forcing treatment to be terminated). Alternatively, the study itself may end. These special situations can be dealt with using a group of statistical models known as survival analyses, in which the dependent variable is time until an event, and participants are computed as people*time.^1^^,^ 2

Survival analysis data can be shown in the form of a survival curve (Kaplan-Meier) or a survival table, which illustrate the fraction of participants remaining under observation as a function of time, i.e. those who did not suffer the event and were not “censored”, which is a term used to denote termination of follow-up (Figure 1). These analyses can be used to estimate parameters such as the time taken to reach a percentage of outcomes and the percentage of events that occur within an interval of time, or to make comparisons between the time taken for events to occur in different subgroups.^3^^-^ 5

**Figure 1:** Survival functions (Kaplan-Meier curves) for cardiovascular events among men over the age of 55 (n = 176) seen at the Hospital das Clínicas, Faculdade de Medicina de Botucatu (Botucatu, SP, Brazil), by presence or absence of Lovibond nail angle abnormality (≥ 180º). Log-rank (p = 0.06); Tarone-Ware (p = 0.05); Gehan-Breslow (p = 0.04); Peto-Prentice (p = 0.04).

As an illustration, consider a cohort of 176 men over the age of 55, followed for 10 years (in an ongoing study), in order to evaluate the occurrence of cardiovascular events (myocardial infarction, angina, claudication, stroke, or arterial revascularization surgery) and their association with skin problems, in this example, flattening of the Lovibond angle (≥ 180º). Over a median (p25-75) follow-up period of 3.2 (2.5-5.0) years, there are 25 events (45%) among participants with the flattened nail angle and 53 events (44%) among those with normal angles (RR = 1.01, 95%CI 0.65 to 1.57; p = 0.95). However, as a function of time, the events occurred earlier among those with the nail abnormality (Figure 1). At 4 years follow-up, observation of half of the cases had already terminated, whereas more than 60% of the controls were still on the study, and the control group only reached 50% survival after 5 years of follow-up. The probability of survival after a given follow-up period or the regularity of mortality rates can also be estimated.

The principal hypothesis tests for inferential comparison between subsets are: the Gehan-Breslow (generalized Wilcoxon) and Peto-Prentice tests, which determine greater weight for higher numbers of cases at risk (events at the start of the observation); the Tarone-Wire test, which weights both the number of cases and the observation period (sensitive to events during the observation period); and the Log-rank (Mantel-Cox) test, in which all observation points have the same weight, favoring differences observed at the end of follow-up.^6^^,^ 7 All of these tests lose power if the ratio of events alternates between groups as time passes (crossed curves).

As illustrated by the example in Figure 1, since there were many censored participants and many events in the first half of follow-up, the Gehan-Breslow, Peto-Prentice, and Tarone-Ware tests all indicated p values ≤ 0.05. In contrast, the Log-rank test was influenced by the second half of the follow-up period, in which a smaller number of cases were still under observation, resulting in borderline significance (p = 0.06). Beyond seeking statistical significance, researchers should be careful in their choice and interpretation of tests, with a view to generalization of the results, not least because absence of events in one of the subsets combined with a high frequency of censoring should be seen as a sign that there may be reasons for the drop outs that are related to the exposure.^5^^,^ 8

In a survival analysis, the effect size between subsets is estimated as a hazard ratio (HR), which can be interpreted as the relative risk of occurrence of the event as a function of time. The HR is calculated using a proportional hazards model (Cox regression), which also enables HR to be adjusted for other covariates (irrespective of the distribution), providing a multivariate analysis of the study.^2^^,^ 9 ^,^ 10

The 95% estimation and p value should both be included when reporting HR. For the example illustrated in Figure 1, after adjustment for age, smoking, dyslipidemia, diabetes, family history, and hypertension, the HR for nail abnormality was 1.7 (95%CI 1.1 to 2.9; p = 0.03). The interpretation of this result is that, in this study population, cardiovascular events occurred 1.7 more quickly among participants with nail abnormalities and that this difference was significant, independently of other classical risk factors.^11^^-^ 13

A condition that must be met for the Cox model to provide adequate performance is parallelism of occurrence of events in the subsets being compared (uniformity of risk as a function of time); if this is not the case, HR will vary in response to follow-up time. The principal method for analyzing parallelism is the Log-Log diagram (Figure 2), which must not show the curves crossing.^2^^,^ 8 ^,^ 11 ^,^ 13

**Figure 2:** Log-Log diagram of survival analysis data for cardiovascular events in men over the age of 55 (n = 176) seen at the Hospital das Clínicas, Faculdade de Medicina de Botucatu (Botucatu, SP, Brazil), by presence or absence of Lovibond nail angle abnormality (≥ 180º).

Very often, the dependent variable in a longitudinal study is recorded as a quantitative parameter (for example, arterial blood pressure, glycemia, or a quality of life index). In these cases, it is necessary to dichotomize or categorize the variables (for example, as hypertensive, diabetic, impact on quality of life, or arterial obstruction < 50%) in order to conduct a survival analysis. The criteria used to choose the cutoff points for categorization have a direct impact on the results, and must be defined with parsimony and scientific plausibility, and should be justified in detail in the methodology. It is also recommendable to analyze the sensitivity of the results, weighting the impacts of different cutoff points on the magnitude of the results, in order to improve the consistency of the conclusions.^14^

The sample size in a longitudinal study employing survival analysis is influenced by follow-up time, number of censored data points, number of subsets for comparison and the total frequency of events and the differences in events between subsets. In general, models do not tend to perform well (large type 2 errors) when there are fewer than 10 events (per analysis subset) and the number of participants is less than 10 per subset. On the basis of these principles, it is advisable to conduct a pre-test with a shortened follow-up time in order to ensure an appropriate sample.^15^^,^ 16 Below we show a formula that can be used to calculate the number of events needed as a function of HR and which is dependent on tolerance of type 1 errors, usually set at two-tailed 5% (Z_α/2_ = 1.96), and type 2 errors, which is usually single-tailed 20% (Z_β_ = 0.84).^16^^,^ 17 The proportion of participants in each subset is represented by p1 and p2.

$$Events={\left(Z_{\alpha /2}+Z_{\beta }\right)}^{2}/p1\times p2\times {\left(\ln\;HR\right)}^{2}$$ (1)

Considering a pre-test with the data from the example in Figure 1, we have two groups, of 120 (68%) and 56 (32%) participants respectively. There were 78 events and HR was calculated as 1.7. Inserting these data into the formula shown above, we have: (1.96 + 0.84)^2^ / 0.32 × 0.68 × (*ln* 1.7)^2^ = 128 events needed. This indicates a need to increase the sample size and/or extend the follow-up period.

Since survival analysis is very sensitive to changes, it must be conducted with the maximum of methodological rigor and it is advisable to have the support of an experienced statistician or epidemiologist. Subset selection biases, different disease durations before enrollment (left censoring), irregularities of randomization, and failure to record or control censoring are examples of methodological issues that can compromise results. There are also special cases, such as comparison of paired data, ordinal factors of comparison (for example, cancer staging), covariates with behavior that changes over time (for example, medication dosages, cholesterol levels), comparison of groups with non-parallel behavior, or recurrent events (for example, re-infection, re-occlusion, re-infarction) which require different models that are beyond the scope of this text.^5^^,^ 8

Finally, choosing a survival analysis technique to evaluate longitudinal data does not rule out using other classic statistical analysis methods in the same study, rather it offers a different perspective on the same phenomenon.^18^
